# In the Same Boat? Health Risks of Water Recreation Are Not Limited to Full-Contact Activities

**DOI:** 10.1289/ehp.120-a77a

**Published:** 2012-02-01

**Authors:** Wendee Holtcamp

**Affiliations:** Houston-based freelancer Wendee Holtcamp has written for *Nature*, *Scientific American*, *National Wildlife*, and other magazines.

Forty years after the Clean Water Act established a goal that the nation’s waters be suitable for recreation, many waterways still fail to meet that standard. Those deemed unsuitable for full-contact recreation are nevertheless used for limited-contact water activities such as boating, kayaking, and fishing. Although several states have started exploring site-specific standards for limited-contact recreation in waterways with high bacterial concentrations, very little is known about whether these activities are safe in such settings. The Chicago Health, Environmental Exposure, and Recreation Study, a prospective cohort study of more than 11,000 users of waterways in and around Chicago, sought to estimate the health risks associated with limited-contact recreation in potentially contaminated waters [*EHP* 120(2):192–197; Dorevitch et al.].

The study compared health outcomes between people who engaged in limited-contact water recreation in the Chicago Area Waterways System (CAWS), those who engaged in limited-contact recreation in “general-use” waters that had been deemed suitable for full-contact recreation, and a reference group of people who engaged in outdoor recreation near but not in contact with water. The CAWS consists primarily of wastewater, including 300 million gallons received daily from each of two wastewater plants. This effluent is treated with an activated sludge process but is not chemically disinfected. Earlier studies showed levels of *Escherichia coli* and *Enterococcus* bacteria were much higher and *Cryptosporidium* and adenovirus type F were detected more frequently in the CAWS than in samples from the general-use waters.

Study participants were interviewed prior to their recreation activity, with followup interviews by phone 2, 5, and 21 days afterward. CAWS users experienced more eye symptoms than those in the reference or general-use water groups, and people who engaged in water recreation activities at all were significantly more likely than the reference group to develop gastrointestinal illness in the first 3 days following recreation. The researchers found no difference in the frequency at which gastrointestinal illness developed between general-use and CAWS users. They speculate that the two groups may have received comparable average doses of ingested pathogens, with CAWS users exposed to waters with higher pathogen densities but general-use users more likely to immerse their heads and faces in the water. There was a higher risk of eye symptoms in CAWS users than in the reference or general-use groups, but no statistical differences existed between the 3 groups for development of respiratory illness, rash, or ear problems.

Strengths of the study include its large size and prospective exposure self-assessment, while limitations include the reliance on self-reported health outcomes and the inability to quantify actual exposures to pathogens during recreational activities. The results suggest that, contrary to general assumptions, there are health risks associated with limited-contact recreation in waterways—even in water bodies designated as safe for swimming and other full-contact activities.

**Figure:**
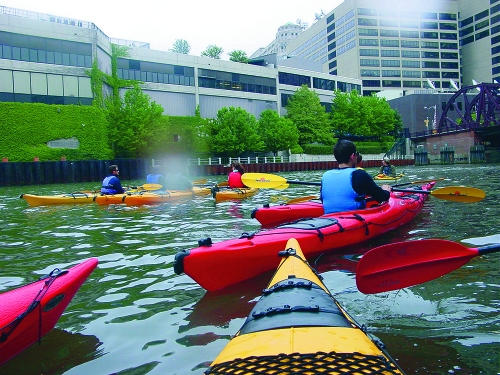
Kayaking on the CAWS, 2009. Even limited-contact water recreation can result in pathogen exposures. © 2012 The Two Boxers

